# ABO subgroup incompatibility with severe hemolysis after consecutive allogeneic stem cell transplantations

**DOI:** 10.1002/jha2.190

**Published:** 2021-03-24

**Authors:** Judith S. Hecker, Adam Wahida, Erik Hameister, Aneta Filo, Jürgen Ruland, Florian Bassermann, Martin Hildebrandt, Mareike Verbeek, Hendrik Poeck

**Affiliations:** ^1^ Department of Medicine III, Hematology and Internal Oncology, TUM School of Medicine Technical University of Munich Munich Germany; ^2^ Torsten‐Haferlach‐Leukemia‐Diagnostics Foundation Munich Germany; ^3^ Institute of Clinical Chemistry and Pathobiochemistry TUM School of Medicine Technical University of Munich Munich Germany; ^4^ Department of Medical Oncology and Hematology University of Zurich and University Hospital Zurich Zurich Switzerland; ^5^ Department of Transfusion Medicine, Cellular Therapy and Hemostaseology University Hospital, LMU Munich Munich Germany; ^6^ Department of Internal Medicine III, Hematology and Internal Oncology University Hospital Regensburg Regensburg Germany

**Keywords:** A1/A2 subtyping of blood group A, ABO incompatibility, allogeneic hematopoietic stem cell transplantation, hemolysis

## Abstract

Allogeneic hematopoietic stem cell transplantations (HSCTs) represent a curative strategy for treating hematologic malignancies yet bear dangerous and frequently life‐threatening complications including the development of graft‐versus‐host disease. Here, we present a case of a patient that suffered from relapsed/refractory multiple myeloma, a hematologic neoplasm characterized by clonal proliferation of malignant plasma cells in the bone marrow. During the course of his disease, the patient underwent consecutive allogeneic HSCTs, during which he developed a clinical meaningful and hitherto unreported ABO subgroup incompatibility, leading to persistent hemolysis. Testing for ABO subgroups during donor selection, especially after consecutive allogeneic HSCTs, may therefore aid to prevent these complications.

## 1 CASE REPORT

**FIGURE 1:**
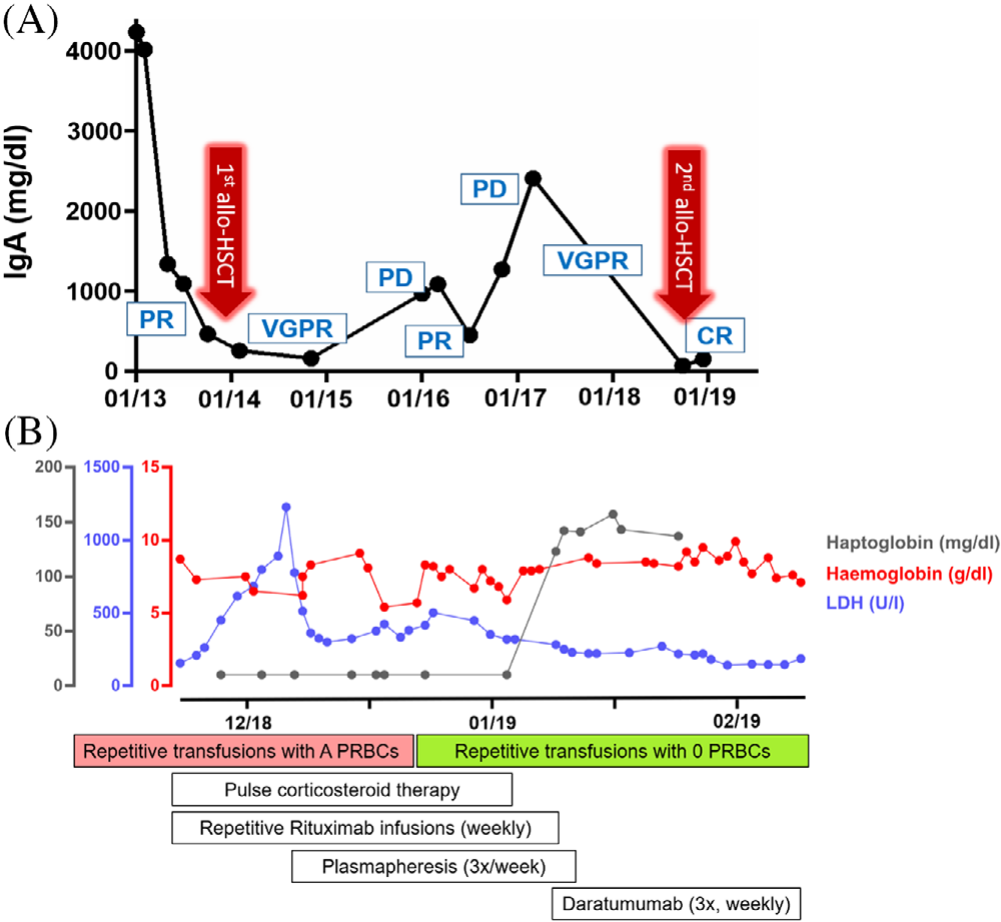
Course of disease and hemolysis. (A) Therapy response including IgA time course in relation to both allogeneic HSCTs from initial diagnosis 2013 to patient death 2019. (B) Time course of hemolysis with clinical laboratory values showing the hemoglobin, haptoglobin, and lactate dehydrogenase levels in serum in relation to the therapeutic algorithm and blood transfusion setting Abbreviations: CR, complete response; LDH, lactate dehydrogenase; PD, progressive disease; PR, partial response; PRBC, packed red blood cells; VGPR, very good partial response.

The patient was initially diagnosed with a standard risk multiple myeloma (MM) in January 2013 (Table 1A). Partial response (PR) was achieved after three cycles of induction therapy with lenalidomide, doxorubicin, and dexamethasone, which was administered within the randomized multicenter phase III trial (DSMM XIV study) by the German Study Group for MM. Upon request of the patient, he was excluded from this study in favor of an intensified induction therapy consisting of bortezomib, lenalidomide, dexamethasone, cisplatin, doxorubicin, cyclophosphamide, and etoposide (VRD‐PACE). This new regimen was followed by high‐dose chemotherapy with melphalan and an autologous hematopoietic stem cell transplantations (HSCT) which resulted in a very good PR (VGPR). Three months later (November 2013), the patient underwent the first allogeneic HSCT from an HLA‐identical sibling stem cell donor (Table 1B). Following engraftment, the patient switched from his initial blood group O Rh^+^ to his sibling's A Rh^–^. Required blood cell transfusions were performed using A Rh^–^ packed red blood cells (PRBCs) without any complications. During the further course, the patient subsequently received maintenance therapy with lenalidomide and remained in VGPR (Figure 1A).

**TABLE 1:** Disease (A), donor and recipient (B) characteristics

A. Disease characteristics		
Disease stage	Initial diagnosis	Progressive disease
Date	January 1st, 2013	December 1st, 2015
(After 1st allogeneic HSCT)		
Ig Subtype	IgA kappa	IgA kappa
Risk profile	Standard	High
International staging system (ISS)	1	Unknown
CRAB features	Osteolytic lesions and soft tissue‐like masses	Osteolytic lesions and soft tissue‐like masses
Durie‐Salmon staging system	IIIA	IIIA
Karyotype	47, XY, +17	51, XY, + 3, + 9, + 11, + 15, + 17

B. Donor and recipient characteristics								
HSCT	Donor	Stem cell source	CD34^+^ cells	Conditioning regimen	Immunosuppressive regimen	Blood group donor	Neutrophil engraftment	Platelet engraftment
1st allogeneic HSCT	HLA‐matched sibling	PB and BM	1.0 x 10^6^/kg	Fludarabine 30 mg/m² (4 days) with melphalan 140 mg/m² (single dose) and ATG 10/20/30 mg/kg (Thymoglobulin, 3 days)	Cyclosporine 5 mg/kg and mycophenolate mofetil 2 g/day	A_1_ Rh^‐^ ccddee kk	Day +15	Day +18
2nd allogeneic HSCT	HLA‐matched unrelated donor	PB	5.5 x 10^6^/kg	Fludarabine 30 mg/m² (4 days) with treosulfan 1400 mg/m² (3 days) and ATG 10/20/30 mg/kg (Thymoglobulin, 3 days)	Cyclosporine 5 mg/kg and mycophenolate mofetil 2 g/day	A_2_ Rh^‐^ ccddee kk	Day +28	Not reached

At the end of 2015, the patient was diagnosed with progressive disease (PD) with increased immunoglobulin A (IgA) levels and multiple new active osteolytic bone lesions as well as expansile lesions with soft tissue masses detected by ^18^F‐FDG‐PET (Table 1A). Moreover, the malignant plasma cells had expanded in the bone marrow and harbored multiple new genetic aberrations, evolving from the known singular trisomy 17 to a complex aberrant karyotype (51, XY, +3, +9, +11, +15, +17). Subsequently, treatment according to the VRD regimen (bortezomib, lenalidomide, dexamethasone) was initiated [1]. Unfortunately, the patient progressed after one cycle of VRD. We therefore escalated the treatment by administering four cycles of the VCD regimen consisting of pomalidomide, bortezomib, cyclophosphamide and dexamethasone accompanied by a total of four doses of donor lymphocyte infusions. This resulted in PR in July 2016, as determined by decreased serum IgA levels.

In November 2016, the patient presented again with PD characterized by multiple new osteolytic lesions and extensive bone marrow expansion of the malignant plasma cells. Despite a renewed escalation of the therapy with combinations of elotuzumab, pomalidomide, and dexamethasone followed by carfilzomib, cyclophosphamide, and dexamethasone, no response could be achieved. Furthermore, the patient also underwent radiation of osteolytic lesions and soft tissue‐like masses for pain management. Finally, a PR was achieved via a combination of daratumumab, pomalidomide, and dexamethasone (eleven cycles from July 2017 until May 2018). Aiming to consolidate the response, the treatment regimen was escalated in June 2018 to venetoclax, carfilzomib, and dexamethasone, which resulted in a VGPR.

In light of the high‐risk disease profile, the patient's young age, and good clinical condition (ECOG 0), a second allogeneic HSCT was considered and after conditioning with fludarabine, treosulfan, and ATG ultimately performed in October 2018 (Table 1B). This resulted in a complete remission (CR) and minimal residual disease negativity (Figure 1A). Administering cyclosporine and mycophenolate mofetil as graft‐versus‐host disease (GVHD) prophylaxis, the patient had no signs or symptoms of acute GVHD (Table 1B).

This second donor was an HLA‐matched unrelated donor bearing blood group A Rh^–^ (Table 1B). The screen for alloreactive antibodies against donor erythrocytes was clouded by ubiquitous positivity due to interferences by previous application of daratumumab. Pre‐treating test erythrocytes, and PRBC samples with dithiothreitol (DTT) improved the diagnostic yield and allowed for conclusive crossmatch results. Following transplantation, transfusions of PRBCs with blood group A Rh^–^ were initially well tolerated. However, after 4 weeks of almost daily PRBC transfusions, the patient developed a Coombs positive hemolysis (direct Coombs test) with persistent haptoglobin levels below 10 mg/dl and elevated lactate dehydrogenase (LDH 300–1228 U/l) (Figure 1B). We next sought to identify the causative alloreactive antibody using a series of detection methods. First, we performed a standard antibody screening assay (gel column agglutination test with three test cells) and secondly with a more elaborate screen test panel (further 27 test cells). Then, we scrutinized our assay for possible interference by daratumumab by performing these assays with and without DTT. However, elution of the antibodies bound to the patient's erythrocytes yielded no specificity in any of the used test erythrocytes at our disposal. We therefore concluded that, with the last dosing of daratumumab 6 months ago, daratumumab was no longer interfering in our assay [2].

We first started to treat the hemolysis with corticosteroids (initial dose of prednisone of 2 mg/kg/day) representing the first‐line treatment for autoimmune hemolytic anemia. Since this did not alter the course of the hemolysis (Figure 1B), we sought to target the antibody‐producing B‐cell populations by weekly administering rituximab (375 mg/m^2^) throughout 4 weeks [3, 4]. After these measures did not yield a sufficient improvement of the hemolysis, we aimed to deplete the antibody reservoir by initiating a 3‐weekly plasmapheresis [5]. Moreover, since all these measures did not bear any sign of success, an off‐label treatment with daratumumab (16 mg/kg, weekly for 3 weeks) was started to specifically deplete the plasma cell pool responsible for production of the alloreactive antibodies [6]. Still, crossmatching of A Rh^–^ PRBCs with the patient's plasma was positive in most, but not all PRBCs tested. Surprisingly, those PRBCs demonstrating a negative crossmatch were all blood group A_2_ Rh^–^ based on a capture solid phase technology. At the same time, we identified an anti‐A antibody in the serum of the patient. Subsequently, we switched to transfusing 0 Rh^–^ PRBCs, which was well tolerated and resulted in a swift return to normal levels of LDH and haptoglobin (Figure 1B). Further investigation into the first donor's blood group revealed that he was A_1_ positive (the most prevalent subgroup in Germany) whereas the second stem cell donor was A_2_ positive (Table 1B) [7].

Immune‐mediated hemolytic anemia is frequently observed and a common complication after allogeneic HSCT [8], especially in the context of passenger lymphocyte syndrome (PLS) comprising hemolysis associated with minor ABO incompatibility between the donor and the recipient (most common A^+^ recipient, O^+^ donor) [9]. PLS usually occurs immediately (between 5 and 15 days) post‐transplant and is generally moderate but has also been described to cause fatal hemolysis and potentially death [9, 10]. The incidence of PLS‐associated hemolysis is waning since anti‐B‐cell immunosuppressive therapy is increasingly administered as a component of GVHD prophylaxis, as conducted on this patient.

Here we present a rare case of severe, late‐onset and treatment‐refractory hemolysis in which B cells from the second allogeneic HSCT donor reacted and produced alloreactive antibodies against the A_1_ epitope of the erythrocytes from donor 1 and therefore administered A_1_ Rh^–^ PRBCs. The hemolysis occurred in coincidence with neutrophil engraftment approximately 4 weeks after the second allogeneic HSCT and persisted until the patient was transfused with O Rh^–^ instead of A Rh^–^ PRBCs. Therefore, we conclude that this course of severe hemolysis may have been prevented and should draw attention to A_1_/A_2_ subtyping of blood group A, especially in case of consecutive allogeneic HSCTs.

During the further course, the patient suffered from a generalized herpes simplex virus (HSV) infection with severe herpetic gingivostomatitis, HSV viremia, and HSV DNA detection in the bone marrow requiring prompt institution of appropriate antiviral therapy. Unfortunately, upon persisting CR and initial neutrophil engraftment (day +28, Table 1B) as well as detection of a complete chimerism, the hematopoietic system of the second donor failed to reconstitute a trilineage of blood cells permanently. Consequently, the inability to mount sufficient immune responses against opportunistic infections and the subsequent development of sepsis led to the death of the patient.

## 2 DISCUSSION

In conclusion, we note that the patient developed a clinically meaningful Coombs positive hemolysis due to the generation of an alloreactive antibody against the A_1_ antigen following a second allogeneic HSCT. Erythrocytes in antibody screen assays are usually O Rh^+^, distracting from the possibility of an alloantibody directed against A_1_. Based upon our case, we speculate that mismatches within the ABO subgroups may play a more significant role in the future with increasing numbers of consecutive allogeneic HSCTs and broadening pools of potential donors [11]. Implementing rigorous blood group testing during the process of donor selection may therefore prevent complications arising from for ABO subgroup incompatibilities, especially after consecutive allogeneic HSCTs.

## 3 METHODS

Blood group determination and serological testing were performed using automated platforms using the solid‐phase technology (ImmucorNeo Galileo analyzer, ImmucorNeo, Dreieich, Germany) and standard gel techniques (IH‐1000, Bio‐Rad, Cressier sur Morat, Switzerland). Additional manual testing included the use of standard gel techniques and commercially available test cell panels (Bio‐Rad, Cressier sur Morat, Switzerland, or Grifols Deutschland GmbH, Frankfurt/M., Germany). Serum and eluate indirect antiglobulin tests and direct antiglobulin test were performed using polyspecific Ig cards. RBC‐bound antibodies were eluted from the cells using an acid‐elution kit (BAG, Lich, Germany).

## CONFLICT OF INTEREST

The authors declare no competing financial interests.

## AUTHOR CONTRIBUTIONS

All authors were involved in the clinical and diagnostic management of the reported patient and contributed to the writing of the manuscript and approved the final version. Conceptualization: Adam Wahida, Erik Hameister, Martin Hildebrandt, Judith S. Hecker and Hendrik Poeck. Methodology: Adam Wahida, Erik Hameister, Aneta Filo and Martin Hildebrandt. Investigation: Judith S. Hecker, Adam Wahida and Erik Hameister. Resources: Judith S. Hecker, Mareike Verbeek, Martin Hildebrandt, Hendrik Poeck, Florian Bassermann and Jürgen Ruland. Writing: Adam Wahida, Erik Hameister, Judith S. Hecker, Hendrik Poeck. Visualization: Adam Wahida and Judith S. Hecker. Supervision: Mareike Verbeek, Martin Hildebrandt and Hendrik Poeck.
